# Recent Topics and Perspectives on Esophageal Cancer in Japan

**DOI:** 10.31662/jmaj.2018-0002

**Published:** 2018-09-28

**Authors:** Masayuki Watanabe

**Affiliations:** 1 Department of Gastroenterological Surgery, the Cancer Institute Hospital of Japanese Foundation for Cancer Research, Tokyo, Japan

**Keywords:** Esophageal cancer, risk factor, genetic background, minimally invasive esophagectomy, immune checkpoint

## Abstract

Despite recent advances in multidisciplinary treatment strategy, outcomes of esophageal cancer treatment still remain unsatisfactory. There are two histologic subtypes of esophageal cancer, namely, squamous cell carcinoma and adenocarcinoma, and these subtypes turned out to be genetically separate diseases. I focused on nine topics among the cancer's epidemiology, diagnosis, and treatment, and reviewed the literature. Although the number of patients with esophageal cancer has been continuously increasing, the cause of esophageal cancer is evident in a substantial proportion of patients, and public education may be able to decrease its incidence. Early detection and less invasive treatment will improve the outcome of patients. Minimally invasive esophagectomy decreased surgical invasiveness and improved short-term outcomes in the clinical trials. Centralization of patients to high-volume centers and introduction of multidisciplinary perioperative care bundle may further improve the outcome of patients undergoing esophagectomy. Although no targeting agent has shown efficacy in patients with esophageal cancer, immune checkpoint blockades are promising, and the results of phase III trials are awaited.

## Introduction

An estimated 455,800 new esophageal cancer cases and 400,200 deaths occurred in 2012 worldwide, and esophageal cancer was the sixth leading cause of cancer-related mortality in males ^(1)^. In Japan, an estimated 22,812 individuals were newly diagnosed with esophageal cancer in 2013 and 11,483 died from this disease in 2016 ^(2)^. The overall 5-year survival rate ranges from 15% to 25% worldwide, despite the recent advances in multidisciplinary treatment strategy ^(3)^. In Japan, the 5- and 10-year survival rates of male esophageal cancer patients were estimated to be 36% and 24%, respectively, while those of female esophageal cancer patients were estimated to be 44% and 32%, respectively ^(2)^.

There are two major histologic subtypes of esophageal cancer, namely, squamous cell carcinoma (SCC) and adenocarcinoma (ADC). Although SCC accounts for approximately 90% of esophageal cancer cases worldwide, the incidence and mortality rates associated with ADC are rising and have surpassed those of SCC in several regions in North America and Europe ^(4)^. In Japan, although SCC still remains the main histologic subtype, the incidence of ADC has been increasing, owing to the decreasing incidence of *Helicobacter pylori* (HP) infection and the subsequent increase in gastroesophageal reflux disease (GERD) ^(5)^.

In this study, I aim to present recent topics in the epidemiology, diagnosis, and treatment of esophageal cancer and discuss the future directions of the management of esophageal cancer patients.

## Materials and Methods

Recent topics of esophageal cancer diagnosis and treatment were reviewed. I focused on nine topics, including risk factors, genetic backgrounds, temporal trends in Japan, advances in diagnostic imaging, endoscopic treatment for early esophageal cancer, minimally invasive esophagectomy, novel surgical approach, adjuvant and neoadjuvant treatment, and immune checkpoint blockades.

## Results

### Risk factors

Cigarette smoking and alcohol consumption are the major risk factors for esophageal SCC. A meta-analysis to evaluate the risk of cigarette smoking for esophageal cancer among Japanese revealed summary relative risks (RRs) of current and former smokers were 3.73 (95% CI, 2.12-6.43) and 2.21 (1.60-3.06), respectively ^(6)^. However, the correlation between the amount of cigarette consumption and risk of esophageal cancer remains unclear, because the information on cigarette consumption was investigated by questionnaire in every study, and a different categorization of smoking exposure was used in each questionnaire. The case-control studies among Japanese revealed that RRs of alcohol were 11.88 (95% CI, 4.41-31.99) at 50 g/day of pure alcohol intake and 33.11 (95% CI, 8.15-134.43) at 100 g/day of pure alcohol intake ^(7)^. The combined effect of tobacco smoking and alcohol drinking is synergistic, and a case-control study revealed that the odds ratio for esophageal cancer was 50.1 for heavy smokers and excessive drinkers compared with those who have no history of exposure to these risk factors ^(8)^. A variant form of the aldehyde dehydrogenase 2 (ALDH2) is common among the East Asian populations. This polymorphism is caused by the substitution of glutamate for lysine at position 487. The lysine allele encodes an inactive protein ^(9)^. Deficiency in functional ALDH2 was found to cause an alcohol-flushing response and increase the risk of alcohol-related esophageal SCC ^(10)^.

GERD and subsequent Barrett's esophagus are the primary risk factors for esophageal or esophagogastric junction (EGJ) ADC. Patients with at least weekly symptoms of GERD were five times more likely to develop esophageal ADC, while those who experience daily symptoms were seven times more likely to develop esophageal ADC, each compared with individuals without symptoms or with less frequent symptoms ^(11)^. Obesity is known as an important risk factor for the development of GERD. Pathophysiological disturbance in obesity include esophageal motor disorders, lower esophageal sphincter abnormalities, a trend toward the development of hiatal hernia, increased intragastric pressure, and increased gastric capacity ^(12)^. Obesity was also reported to be associated with a higher risk of esophageal and gastric cardia ADC ^(13)^. A meta-analysis revealed that a high body mass index (>25 kg/m^2^) was associated with an increased risk of esophageal ADC, with odds ratios of 2.2 for men and 2.0 for women ^(14)^. The RRs of esophageal or gastric cardia ADC were 2.32 for current smokers and 1.62 for ex-smokers, compared with never-smokers ^(15)^. On the contrary, a meta-analysis did not provide definite evidence of the association between alcohol drinking and esophageal ADC risk ^(16)^. HP infection was reported to decrease the risk of esophageal ADC by 41% ^(17)^, by promoting gastric atrophy, which leads to acid reduction.

### Genetic backgrounds

The findings of the Cancer Genome Atlas project suggested that esophageal SCC and ADC are two separate diseases ^(18)^. Esophageal SCC resembled more of an SCC in the head and neck region than an esophageal ADC, whereas esophageal ADC strongly resembled the chromosomal instability variant of gastric ADC. The most commonly mutated genes in both histologic subtypes were *TP53* and *PIK3CA* ^(19), (20)^. By contrast, *NFE2L2*, *MLL2*, *ZNF750*, *NOTCH1,* and *TGFBR2* were frequently mutated in SCC ^(19)^, while *CDKN2A, ARID1A, SMAD4,* and *ERBB2* were significantly mutated in ADC ^(20)^. In a Japanese population, many SCCs contained mutation in genes that regulate the cell cycle (*TP53*, *CCND1*, *CDKN2A*, and *FBXW7*), epigenetic processes (*MLL2*, *EP300*, *CREBBP*, and *TET2*), and the NOTCH (*NOTCH1* and *NOTCH3*), WNT (*FAT1*, *YAP1*, and *AJUBA*), and receptor-tyrosine kinase phosphoinositide 3-kinase signaling pathways (*PIK3CA*, *EGFR*, and *ERBB2*) ^(21)^. Based on mutational signatures, Japanese SCC patients were assigned to three groups that were associated with environmental (drinking and smoking) and genetic (polymorphisms in *ALDH2* and *CYP2A6*) factors. A cluster with a relatively high mutation rate, which is characterized by C to G/T substitutions with a flanking 5' thymine (the APOBEC signature), was tightly associated with environmental risk factors, including drinking and smoking ^(21)^. However, the correlation between the genetic changes and prognosis of esophageal cancer remains unclear. A clustered abnormality in copy number was observed in several genes, including *CCND1* and *SOX2* and/or* TP63*, in esophageal SCC, whereas a more widespread genomic instability and total DNA copy number alterations were observed in esophageal ADC ^(22)^. Amplification/overexpression of the *ERBB2* gene in esophageal or EGJ ADC were more frequent than that observed in gastric cancer, accounting for 24%-32% ^(23)^.

### Temporal trends in Japan

**Figure 1.**
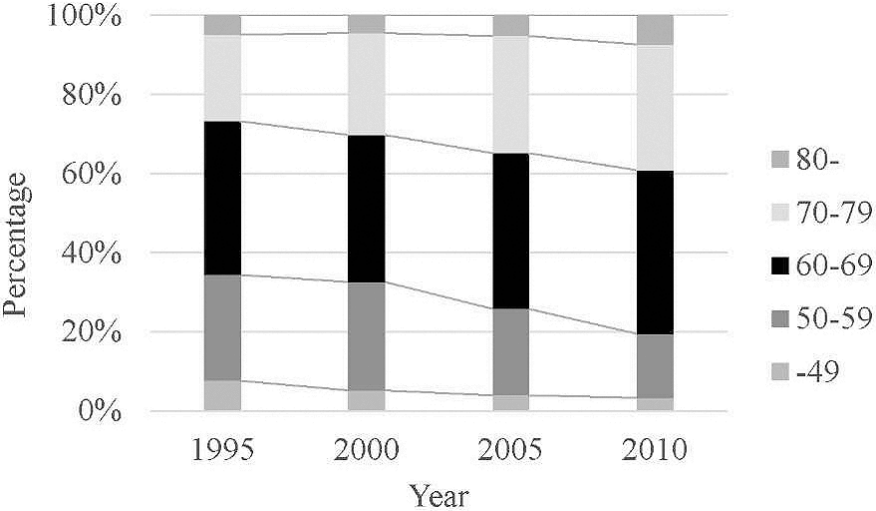
Changes in the age group of patients with esophageal cancer treated in Japan.

In Japan, the number of patients with esophageal cancer has increased every year, and the number of patients who were newly diagnosed with esophageal cancer in 2013 was four times more than that observed in 1975 ^(2)^. The two possible reasons for this increase are as follows: One is the progress in the diagnostic imaging, especially in endoscopic technology, and the other is the rapidly aging society in Japan. Most of the patients with early esophageal cancer have no symptoms and are diagnosed by screening endoscopy. The increase in the number of patients diagnosed with early esophageal cancer contributed to the increase in the number of cases. Meanwhile, as esophageal cancer frequently occurs in the elderly, the number of esophageal cancer patients increases with the aging of society. Figure 1 shows the changes in the age group of patients with esophageal cancer treated in Japan, which was created from the serial nationwide registries conducted by the Japan Esophageal Society ^(24), (25), (26)^. Although the fact remains that esophageal cancer is frequently diagnosed among people in their 60s, the prevalence of esophageal cancer among patients aged 70 years or older has been continuously increasing. In response to the increase in the elderly patient population, we have to establish less invasive treatment strategies for esophageal cancer.

**Figure 2.**
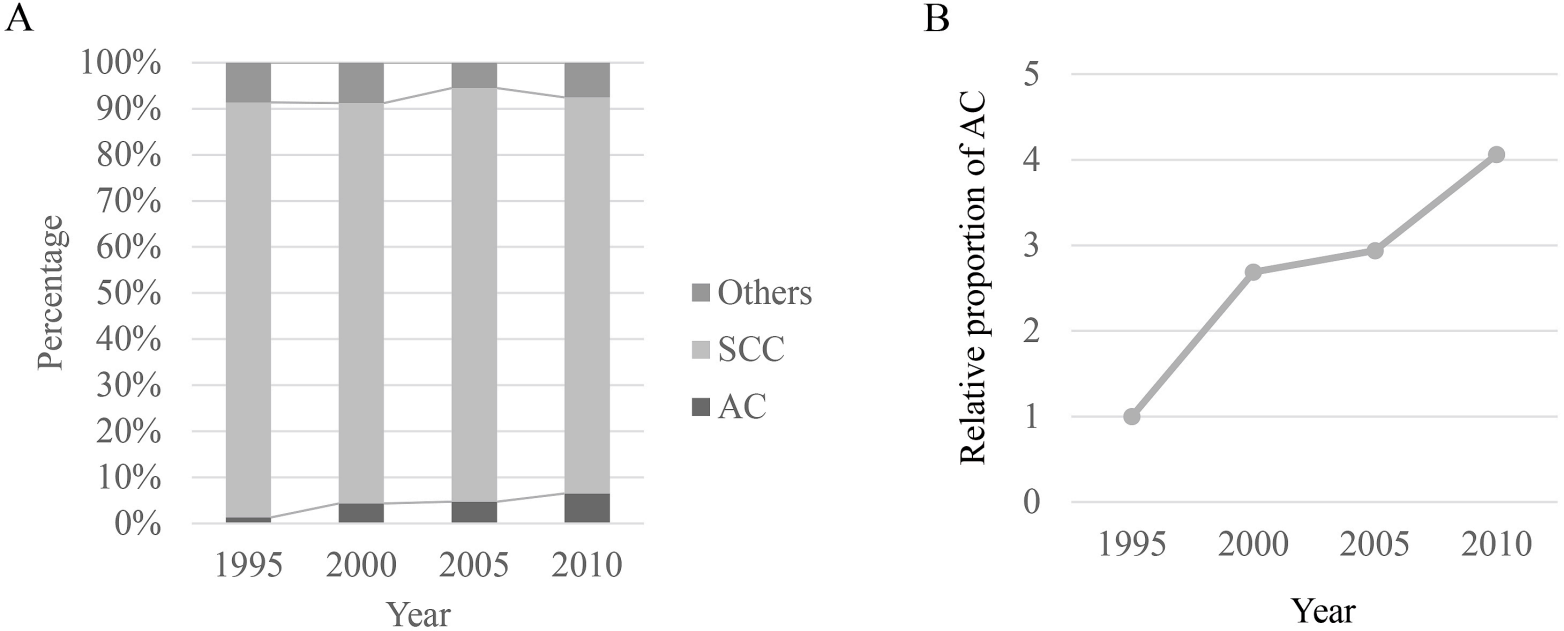
A. Temporal trends of histologic subtypes of esophageal cancer in Japanese patients undergoing esophagectomy, B. Increase in the relative proportion of esophageal ADC.

Increase in the incidence of ADC is another problem in Japan. ADC of the esophagus or the EGJ mainly occurs from Barrett's esophagus, which is pathologically characterized by columnar metaplasia due to GERD. In Japan, because of the high prevalence of HP infection, GERD, and subsequent Barrett's esophagus used to be rare diseases. However, the HP prevalence rate has dramatically decreased, and people born after 1998 appear to have a prevalence of less than 10% ^(5)^. In addition, obesity has become a major health-related problem in Japan. A study conducted in a Japanese adult population who visited a medical center for annual medical check-ups revealed that metabolic syndrome was a reliable predictive factor for the prevalence of GERD ^(27)^. Figure 2A shows the temporal trends of the histologic subtypes of esophageal cancer in Japanese patients who underwent esophagectomy, based on the data of serial nationwide registries ^(24), (25), (26)^. Although SCC remains the main histologic subtype, the prevalence of ADC has been gradually increasing. The proportion of ADC among patients who underwent esophagectomy in 2010 is four times more than that in 1995 (Figure 2B).

### Advances in diagnostic imaging

Endoscopy plays an important role in the early detection of esophageal cancer. Lugol chromoendoscopy is the standard method for detecting early esophageal SCC ^(28)^. The pink color-sign, which is a pink color change observed in the Lugol-unstained area 2-3 min after Lugol staining, has demonstrated sensitivity and specificity of 91.9% and 94.0%, respectively, in the diagnosis of SCC ^(29)^. Narrow-band imaging (NBI), which is a kind of image-enhanced endoscopic technology, can visualize thin blood vessels, such as capillaries, in the epithelium or mucosal layer using two narrow-band illuminations of 415 nm and 540 nm ^(30)^. A prospective randomized trial revealed that NBI detected superficial esophageal SCC more frequently than white-light imaging (97% versus 55%, P < 0.001) ^(31)^. Magnifying endoscopy combined with NBI was used to clearly observe the microvessel morphology and predict the histological invasion depth of superficial esophageal SCC ^(32)^. Recently, the Japan Esophageal Society developed a simplified magnifying endoscopic classification for estimating the invasion depth of superficial esophageal SCC ^(33)^.

Endoscopic ultrasonography (EUS) provides detailed information on the esophageal wall as well as the nodal status. The implication of EUS seems to differ between Japan and Western countries. In Japan, EUS is mainly performed to estimate the subclassification of superficial cancers, which is an important piece of information to determine the indication for endoscopic resection (ER). In contrast, EUS is mainly used to diagnose the invasion depth of advanced cancers as well as assess the nodal status in Western countries. The National Comprehensive Cancer Network (NCCN) guidelines in the United States recommend EUS for cancer staging prior to any treatment ^(34)^.

Contrast-enhanced computed tomography (CT) is the gold standard for esophageal cancer staging. However, lymph node metastasis is diagnosed by CT based on the size, morphology, and contrast medium enhancement of the lymph nodes, and the accuracy remains unsatisfactory. ^18^F-fluorodeoxyglucose-positron emission tomography (FDG-PET), which can detect increased glucose metabolism associated with cancer, improved the accuracy of diagnosing lymph node metastasis ^(35)^. In addition, FDG-PET was more accurate in detecting distant metastasis than CT, and therefore can prevent unnecessary surgical exploration in patients with M1 disease ^(36)^.

### Endoscopic treatment for early esophageal cancer

ER is a less invasive treatment for early esophageal cancer compared with esophagectomy. There are two widely accepted ER methods, including endoscopic mucosal resection (EMR) and endoscopic submucosal dissection (ESD). Although EMR is a safe and effective treatment for early esophageal cancer, a high local recurrence rate has been reported in lesions exceeding 20 mm and in lesions treated by piecemeal resection in SCC ^(37)^. ESD improved the en bloc resection rate and reduced the risk of local recurrence with safety equal to that of EMR ^(38)^. Nationwide data on complications after esophageal ESD in Japan revealed that perforation or perforation-related complications were observed in 422 of 12,899 ESD procedures (3.3%) ^(39)^. Among these 422 cases, 7 (1.7%) underwent open thoracotomy to treat the esophageal perforation. A lower hospital volume and the female gender were factors associated with a higher occurrence of perforation in the study. Bleeding during ESD can be managed by endoscopic closure with endoclips, and delayed bleeding is rarely observed. Esophageal stricture can be clinically problematic, especially when the mucosal defect after ESD exceeded 3/4 of the circumference. Both local (endoscopic injection) and systemic (per oral) prophylactic use of steroids has shown the most consistent and promising results with minimal complications for prevention of esophageal stricture after ESD ^(40)^.

The indication for ER is decided primarily based on the risk of lymph node metastasis. Carcinoma in situ (T1a-EP) and SCCs invading the lamina propria mucosae (T1a-LPM) have lesser possibility of nodal metastasis, and these lesions are absolutely indicated for ER. The nodal metastasis rates of SCCs invading the muscularis mucosa (T1a-MM) and those with slight submucosal invasion (T1b-SM1: < 200μm below the muscularis mucosa) were 9.3% and 19.6%, respectively ^(41)^, and these lesions are relative indications for ER. A detailed pathological study on surgically resected specimens of superficial esophageal SCC revealed that the lymph node metastasis rates of T1a-MM tumors were 10.3% (4/38) and 41.7% (5/12) with and without lymphovascular involvement (LVI), respectively ^(42)^. Although it remains controversial if additional treatment should be performed in patients with T1a-MM tumors without LVI, additional treatment should be recommended when the pathological diagnosis reveals T1b tumors or positive ly.

There is no consensus on the indication for ER in esophageal ADC, because little is known about the risk of lymph node metastasis in this disease. In clinical practice, the indication for ER in esophageal ADC is decided based on the criteria for diagnosis and treatment of SCC. Recently, a multicenter retrospective study conducted in 13 high-volume centers in Japan revealed that ly, a poorly differentiated component, and a lesion size > 30 mm were the independent risk factors for lymph node metastasis in superficial esophageal ADC ^(43)^. They also found that patients with mucosal and submucosal cancers (1-500 μm invasion) without these risk factors have a low incidence of developing lymph node metastasis.

### Minimally invasive esophagectomy

Although esophagectomy remains the mainstay for curative-intent treatment of esophageal cancer, it is a highly invasive surgery and is associated with significant morbidity and mortality rates. Minimally invasive esophagectomy (MIE) using thoracoscopic and/or laparoscopic approaches has been developed to minimize both surgical injury and invasiveness. The TIME trial is the only randomized controlled trial that compared the short-term outcome of MIE and open esophagectomy (OE) ^(44)^. The primary outcome of the study was pulmonary infection within the first 2 weeks after surgery and during the entire hospital stay, and the secondary outcomes included hospital stay and quality-of-life (QOL) scores. The incidence of pulmonary infection in the MIE group was significantly lower than that in the OE group. In addition, patients who underwent MIE had a shorter hospital stay and better short-term QOL than those who had OE. The MIRO trial compared the short-term outcomes between laparoscopic gastric mobilization and open Ivor-Lewis esophagectomy ^(45)^. A major postoperative morbidity was observed in 64.4% of patients in the open group and 35.9% of those in the MIE group (P=0.0001). Furthermore, major pulmonary complications occurred in 30.1% of patients in the open group and 17.7% in the MIE group (P=0.037). The Eastern Cooperative Oncology Group conducted the E2202 study, which was a prospective phase II multicenter trial, to evaluate the feasibility of MIE ^(46)^. This study revealed a 30-day mortality rate of 2.1%, and MIE was considered to be feasible and safe.

Although several single-center series suggest that MIE can be performed safely and may reduce postoperative morbidity, these findings originate from high-volume centers and does not necessarily reflect the status of the general population. Table 1 summarizes the results of population-based studies comparing the short-term outcomes of MIE and OE ^(47), (48), (49), (50), (51), (52), (53)^. In Japan, the short-term outcomes were also compared between MIE and OE using the National Clinical Database ^(48), (52)^. The incidence of pneumonia was comparable between MIE and OE, whereas that of anastomotic leak was higher and that of surgical site infection was lower in MIE than in OE. The reoperation rates after MIE were higher than those after OE. Mortality rates were comparable between MIE and OE, and hospital stay after MIE was shorter than that after OE.

**Table 1.** Comparison of Short-term Outcomes between Minimally-invasive Esophagectomy and Open Esophagectomy in Population-based Studies.

Authors, Year	Database*	No. of cases**		Morbidity Pneumonia		Leak		SSI		Reoperation within 30 days		Mortality		Hospital stay	
				%	*P*	%	*P*	%	*P*	%	*P*	%	*P*	Days	*P*
Mamidanna R, et al., 2012	HES	MIE	1155	19.9	0.30	NA		NA		8.8	<0.001	4.0	0.61		
		OE	6347	18.6						5.6		4.3			
Takeuchi H, et al., 2014	NCD	MIE	1751	15.0	0.60	14.9	0.016	7.8	0.87	8.0	0.001	3.0	0.26	NA	
		OE	3603	15.5		12.5		7.7		5.6		3.6			
Thirunavukarasu P, et al., 2016	NCDB	MIE	997	NA		NA		NA				3.3	0.17	NA	
		OE	3050									4.3			
Sihaq S, et al., 2016	STS	MIE	814	NA		NA		2.3	<0.001	9.9	<0.001			9	<0.001
		OE	2356					6.6		4.4				10	
Seesing MFJ, et al., 2017	DUGC, PSM	MIE	433	35.6	0.67	21.2	0.028	3.9	0.43			4.7	0.21	13	0.001
		OE	433	34.2		15.5		5.1				3.0		14	
Takeuchi H, et al., 2017	NCD, PSM	MIE	3515	13.9	0.16	12.8	0.86	6.7	0.032	7.0	0.004	2.5	0.41	NA	
		OE	3515	15.2		12.7		8.1		5.3		2.8			
Kauppila JH, et al., 2018	Finland & Sweden	MIE	217	NA		NA		NA				4.1		15	
		OE	1397									6.8		16	

*HES, the Hospital Episode Statistics; NCD, the National Clinical Database; NCDB, the National Cancer Database; STS, the Society of Thoracic Surgeons; DUGC, the Dutch Upper Gastrointestinal Cancer Audit; PSM, propensity score matching. **MIE, minimally-invasive esophagectomy; OE, open esophagectomy. NA, not assessed.

Little is known about the oncologic safety and long-term survival after MIE. A 3-year follow-up of the TIME trial revealed that there was no difference in the disease-free and overall survival rates between the MIE and OE groups ^(54)^. Long-term survival rates were also comparable between the MIE and the OE groups in some of the population-based studies ^(53), (55)^. A 3-year follow-up of the MIRO trial revealed that there was a trend toward improved overall survival and disease-free survival in the hybrid MIE group (67.0% versus 55%, P=0.05; 57% and 48%, P=0.15) ^(56)^.

### Novel surgical approach

Although transhiatal esophagectomy is an established surgical procedure that can reduce early postoperative complications ^(57)^, a major weak point of this procedure is the incomplete mediastinal lymph node dissection. Recently, it was reported that an en bloc lymphadenectomy method in the upper mediastinum using a cervical approach with a single-port mediastinoscopic technique overcomes the weak point when combined with laparoscopic transhiatal esophagectomy with en bloc lymphadenectomy in the middle and lower mediastinum ^(58)^. This technique was recently approved by the National Health Insurance system in Japan. The safety and efficacy of a robot-assisted transhiatal approach for the nontransthoracic radical esophagectomy was also reported ^(59)^.

### Adjuvant and neoadjuvant treatment

Although surgical resection remains the mainstay of esophageal cancer treatment, the long-term outcomes of patients treated with surgery alone are unsatisfactory. To improve the outcomes, trials on adjuvant or neoadjuvant therapy have been performed. Table 2 shows the trials that were used as the basis for the current standard treatment ^(60), (61), (62), (63), (64)^.

**Table 2.** Clinical Trials Influenced the Standard Treatment.

Study name (year)	Histologic subtype*	Treatment arms**	Main results***	
JCOG9204 (2003)	SCC	Surgery alone	5y-DFS 45%	*P* = 0.037
		Surgery + CF	5y-DFS 55%	
MAGIC (2006)	Esophagogastric AC	Surgery alone	5y-OS 23%	*P* = 0.009
		ECF + Surgery + ECF	5y-OS 36%	
JCOG9907 (2012)	SCC	Surgery + CF	5y-OS 43%	*P* = 0.04
		CF + Surgery	5y-OS 55%	
CROSS (2012)	SCC, AC	Surgery alone	Median OS 24.0M	*P* = 0.003
		CRT + Surgery	Median OS 49.4M	
FLOT (2017)	Esophagogastric AC	ECF or ECX + Surgery + ECF or ECX	Median OS 35M	*P* = 0.012
		FLOT + Surgery + FLOT	Median OS 50M	

*SCC, squamous cell carcinoma; AC, adenocarcinoma. **CF, cisplatin+5-fluorouracil; ECF, epirubicine+cisplatin+5-fluorouracil; ECX, epirubicine+cisplatin+xeloda; CRT, chemoradiotherapy; FLOT, 5-fluorouracil/leucovolin+oxaliplatin+taxotere ***DFS, disease-free survival; OS, overall survival.

The standard treatment for SCC differs between Japan and Western countries. The Japan Clinical Oncology Group (JCOG) 9204 study revealed that postoperative chemotherapy using cisplatin and 5-fluorouracil (CF) improved the disease-free survival of patients with node-positive clinical stage II/III SCC ^(60)^. The JCOG 9907 study clarified that the overall survival of patients treated with neoadjuvant CF followed by surgery was significantly better than that of patients treated with surgery followed by CF ^(62)^. Based on these findings, the current standard treatment for clinical stage II/III esophageal SCC is neoadjuvant chemotherapy followed by esophagectomy. The Dutch phase III CROSS trial demonstrated a significant survival benefit with the addition of neoadjuvant chemoradiotherapy (CRT) to surgery in both histologic subtypes ^(63)^. Based on the findings, both the NCCN guidelines and the European Society for Medical Oncology (ESMO) guidelines recommend neoadjuvant CRT followed by surgery for both histologic subtypes.

Meanwhile, in Europe, the MAGIC trial demonstrated a significant survival benefit of perioperative chemotherapy, which consisted of three preoperative and three postoperative cycles of epirubicin, CF, plus surgery, over surgery alone in patients with esophagogastric ADC ^(61)^. The FLOT4 trial, which is a multicenter, randomized phase III study, compared perioperative chemotherapy consisting of docetaxel, oxaliplatin, and fluorouracil/leucovorin (FLOT) with ECF/epirubicin, cisplatin, and Xeloda (ECX) ^(64)^. FLOT significantly improved patients' progression-free survival and overall survival compared with ECF/ECX and is expected to play a role as a key regimen for perioperative chemotherapy.

### Immune checkpoint blockades

The development of immune checkpoint blockades has launched a new era of cancer immunotherapy and changed the overall landscape of cancer treatment ^(65)^. Immune checkpoint blockades, including anti-programmed death-1 (PD-1)/programmed death-ligand 1 (PD-L1) therapies, are considered to be one of the most promising immunotherapy approaches. A meta-analysis revealed that PD-L1 overexpression was found in 43.7% (1,258 of 2,877) of patients with esophageal SCC and high PD-L1 expression was significantly associated with poor overall survival ^(66)^. Pembrolizumab, an anti-PD-1 antibody, was active in pretreated esophageal cancer patients with PD-L1 expressing tumors (> 1% PD-L1 positive tumor cells and/or tumor stroma), with a partial response (PR) rate of 30.4% ^(67)^. A phase II trial of nivolumab, another anti-PD-1 antibody, for patients with pretreated esophageal SCC, demonstrated its efficacy with a PR rate of 15.6% and a complete response rate of 1.6%, and the median overall survival was 12.1 months in 64 evaluable patients ^(68)^. Phase III trials of each drug are ongoing (Nivolumab, 2^nd^ line (NCT02544737) and adjuvant (NCT02743494); Pembrolizumab, 2^nd^ line (NCT02564263)).

Pembrolizumab monotherapy demonstrated promising activity and manageable safety in patients with advanced gastric or EGJ cancer who had previously received at least two lines of treatment ^(69)^. Based on the findings, the United States Food and Drug Administration approved pembrolizumab for the treatment of patients with PD-L1 positive recurrent or advanced gastric or EGJ ADC. In contrast, a randomized, double-blind, placebo-controlled, phase III study revealed that nivolumab provided a survival benefit in patients with advanced gastric or EGJ cancer refractory to, or intolerant of, at least two previous chemotherapy regimens ^(70)^.

## Discussion

Although esophageal cancer is a life-threatening disease, the cause of this disease is evident in a substantial proportion of patients. Therefore, public education may be able to decrease the incidence of esophageal cancer. Especially, approximately 40% of Japanese individuals shows alcohol-flushing response and are at high risk of esophageal SCC from habitual alcohol consumption. Informing ALDH2-deficient young people of their risk of esophageal cancer from alcohol drinking represents a valuable opportunity for cancer prevention ^(71)^. Meanwhile, the increase in esophageal ADC will become a major problem in the HP-negative era. Diagnosis and treatment of GERD may help to prevent Barrett's esophagus and esophageal ADC. In addition, as the standard treatment strategy for esophageal ADC has not yet been established in Japan, clinical trials of ADC in Japanese patients should be conducted.

An aging of esophageal cancer patients is another major problem for clinicians. The reason why esophageal cancer frequently occurs in the elderly remains unknown. As the APOBEC signature related to cigarette smoking and alcohol drinking is one of the characteristics of genetic alteration in esophageal SCC ^(21)^, an accumulation of genetic alterations due to longer exposure to the risk factors may be the cause of esophageal cancer in the elderly. In our experience, ER can be safely performed for the majority of elderly patients. Therefore, early detection of esophageal cancer is very important to cure elderly patients. In contrast, curative-intent treatment for advanced-stage esophageal cancer is highly invasive and is often intolerable in the elderly with several comorbidities. Treatment strategy in the elderly is determined by the performance status, nutritional status, and comorbidity. Although several geriatric assessment scores have been reported ^(72)^, neither the score appropriate for the decision of esophageal cancer treatment nor the strategy based on the score have yet been established.

MIE has improved the short-term outcomes in several clinical trials, but the population-based studies have not yet proven its efficacy. It may be due to the lack of quality control of MIE. In Japan, an analysis of the National Clinical Database revealed that high-volume hospitals (≥ 30 esophagectomies per year) had lower risk-adjusted 30-day and operative mortality rates following esophagectomy compared with low-volume hospitals (< 10 esophagectomies per year) ^(73)^. Centralization of esophagectomy to the high-volume hospitals may contribute to improve the outcomes. We have reported that the introduction of multidisciplinary perioperative management team significantly decreased the incidence of postoperative complications, especially pneumonia, after esophagectomy ^(74)^. The multidisciplinary care bundle may further decrease the incidence of postoperative complications after esophagectomy.

There are only a few cytotoxic drugs that are available for treatment of esophageal SCC. Although molecular targeting agents remarkably improved the outcomes of several types of cancers, no agent has shown efficacy in patients with esophageal SCC. Several next-generation sequencing studies have not detected driver gene mutations in esophageal SCC, whereas the somatic mutation rates were relatively high compared to other solid tumors ^(18), (19), (21)^. Because an increase in the burden of nonsynonymous mutations in tumors has been associated with improvements in response to immune checkpoint blockades ^(75)^, the results of phase III trials are strongly awaited.

In conclusion, the number of patients with esophageal cancer is increasing, and the clinical pictures are changing. Public education may contribute to decrease the incidence of this disease. Early detection of esophageal cancer provides less invasive and effective treatment. A multidisciplinary approach, including perioperative treatment using immune checkpoint blockades, less invasive technique such as ESD and MIE, and perioperative care bundle will improve both short- and long-term outcomes of patients.

## Article Information

### Conflicts of Interest

None

### Approval of Institutional Review Board

This manuscript is a narrative review and does not need the approval.
